# Experiences of families using an early example of a neighbourhood multidisciplinary care team for children and young people in Birmingham, UK: a qualitative exploration of acceptability

**DOI:** 10.3389/fped.2026.1896606

**Published:** 2026-08-17

**Authors:** I. Litchfield, F. Dutton, L. Harper, S. Kaur, C. Luxmore, L. Rahman, C. Wolhuter, C. Bird

**Affiliations:** 1 Department of Applied Health Research, University of Birmingham, Birmingham, United Kingdom; 2 Birmingham Health Partners, Birmingham, Birmingham, United Kingdom; 3 Small Heath Medical Practice, Birmingham, United Kingdom; 4 GreenSquareAccord, Birmingham, United Kingdom; 5 Emergency Department, Birmingham Women’s and Children’s NHS Foundation Trust, Birmingham, United Kingdom; 6 Nuffield Department of Primary Care Health Sciences, University of Oxford, Birmingham, United Kingdom

**Keywords:** child health, health policy, health services research, neighbourhood care, qualitative research

## Abstract

**Introduction:**

In the United Kingdom, the National Health Service is attempting to address the increasing prevalence of chronic conditions, including obesity, and mental ill health among children and young people (CYP) by creating neighbourhood multi-disciplinary teams (NMDTs). These challenges to the health and well-being of CYP are particularly pronounced amongst underserved populations and it is expected that combining health services and social care services can help address these ongoing inequalities. Despite this significant shift in the delivery of care, there is a lack of robust evidence on families’ experiences of these integrated services to inform their development. This work helps address this by providing a qualitative exploration of the acceptability to families of an early example of an NMDT for CYP, the “Sparkbrook Children’s Zone”, as delivered in Birmingham, UK.

**Methods:**

The study used data collected from two focus groups conducted with eight parents whose children had been treated by the Sparkbrook Children’s Zone. The data were analysed using a directed content analysis to populate the relevant domains of Sekhon’s theoretical framework of acceptability.

**Results:**

In summary (by domain), we found that that individuals' awareness of the service was derived from a range of sources and, although understanding that the service was multidisciplinary, they were sometimes unsure of the precise organisations involved (*Intervention coherence*). Parents described how access was supported by a locally situated and collocated service (*Burden*), the importance of the personalised relationship they developed with providers (*Cultural sensitivity*), and finally, the benefits of extended consultation time and the support they received for the family's complex clinical and social needs (*Perceived effectiveness*).

**Discussion:**

Parents appeared to be strongly in favour of the collocated multi-disciplinarity of the service over their usual primary care. However, more work was needed with larger sample sizes, a broader range of demographies, and across services that offered alternative staffing models. In this way, service leads and commissioners could better understand the key characteristics of NMDTs that influenced patient and family acceptability and engagement.

## Highlights

- In the United Kingdom, the rapid implementation of multidisciplinary primary care for children and young people has begun despite a lack of robust evidence.
- This study provides valuable insight into the experiences of underserved families using an early example of a neighbourhood multidisciplinary care team in a socio-economically challenged ward in Birmingham.
- The collocated combination of clinical and social support was welcomed, as was the personalised nature of the clinical care and the value of the practical support and advocacy for families' complex social needs.

## 1 Introduction

Children, young people (CYP), and their families living in high-income countries face mounting challenges to their health and well-being, as the prevalence of chronic conditions, including obesity and mental ill health, continues to increase (1). These challenges are exacerbated in underserved populations, i.e., minoritised and economically deprived communities (2, 3), by a range of socio-economic and cultural pressures that inhibit access to and utilisation of primary or preventive healthcare services (4, 5). This has led to a rise in children's attendances at emergency departments, frequently due to conditions that could be more effectively treated in community settings (6, 7). The need for more responsive, culturally sensitive care has led to efforts in North America, Europe and Australia to prioritise localised service delivery that integrates multiple strands of health and social care and places a greater emphasis on public and preventive health (8–14).

**Figure 1:**
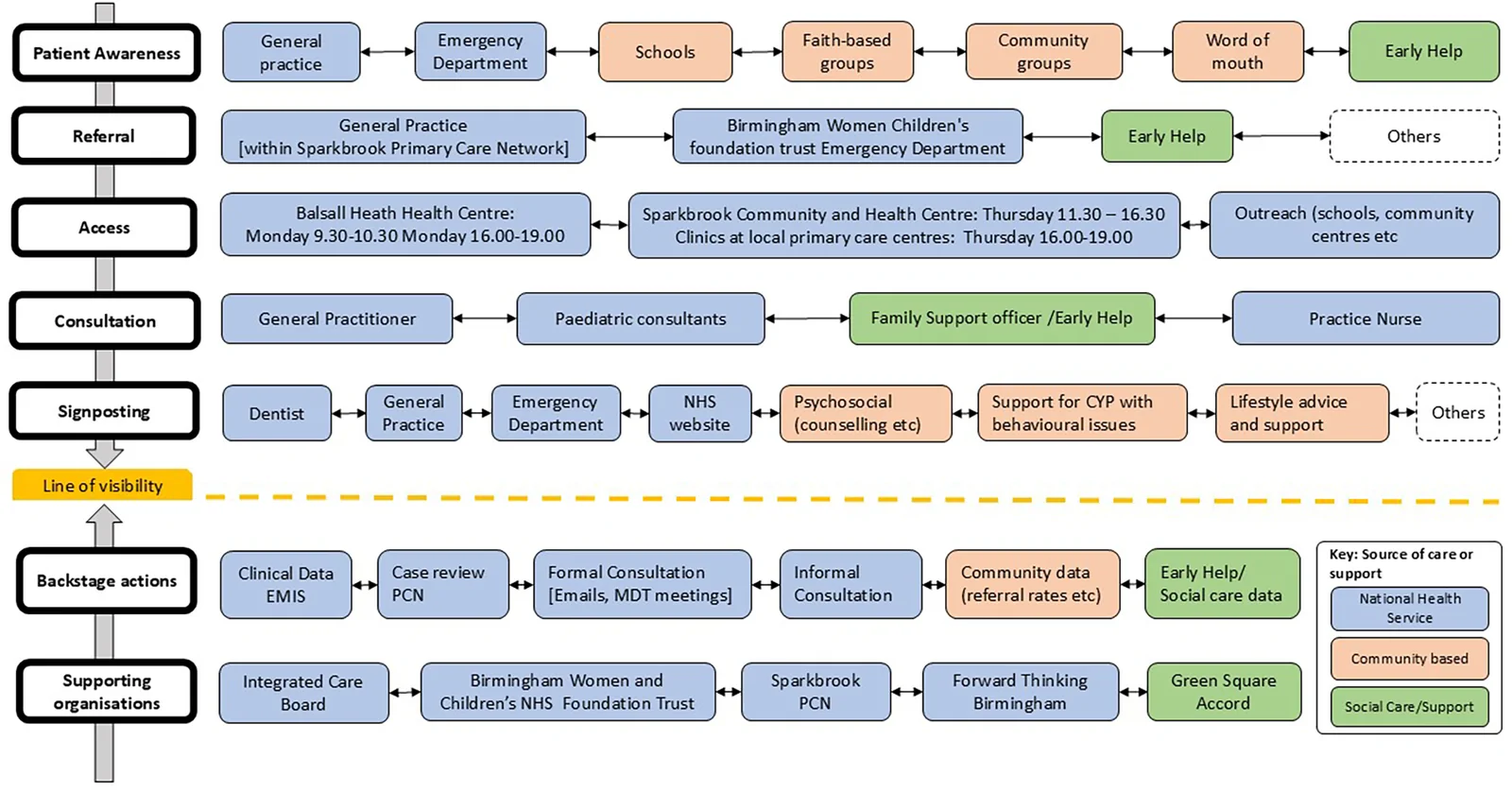
Sparkbrook Children’s zone service blueprint [after (26, 27)].

Policymakers and commissioners in the United Kingdom also recognise that a more holistic approach is needed to address the ongoing challenges faced by CYP, particularly those in underserved populations (12, 15). The most recent National Health Service (NHS) Long Term Plan has attempted to meet these challenges by introducing neighbourhood multi-disciplinary teams (NMDTs) (16). These teams are led by primary care organisations but are integrated with wider services, including education, social support (including social care), and the Voluntary Community, Faith and Social Enterprise (VCFSE) sector (17, 18). It is hoped that grounding these services in the communities they serve will improve their understanding of social, cultural, and environmental contexts and increase engagement and access, leading to a reduction in referrals to secondary care and increased opportunity for early intervention and preventive health (16). Despite this significant shift in the model of delivery, a recent systematic review uncovered few examples of relevant models of localised multidisciplinary care with poorly described methodologies to inform future iterations (19), including how they might be shaped to support the access to and engagement of diverse patient populations (19). One early example of an NMDT that offers this opportunity is the Sparkbrook Children's Zone. The service collocates multiple service elements across two community care centres in a low-income area of Birmingham, UK, to deliver place-based health and social care to underserved CYP (20). The workforce consists of two consultant paediatricians, provided by Birmingham Children's Hospital, each able to offer a half-day a week to manage and run clinics (21); general practitioners (GPs) with an interest in child health; a paediatric nurse to help improve the preventive health offer and perform outreach work, e.g., taking preventive health elements into the community via schools, faith centres, and other local settings; family support workers from an organisation called GreenSquareAccord (22); and a mental health drop-in service provided by PAUSE (23). The clinic was run from two locations, offering afternoon and twilight sessions 2 days a week, was free at the point of care, and accessed via referral from the patient's usual GP. The service blueprint is provided in Figure 1 (24), and a further breakdown of the key components of the service is described in Supplementary File 1.

Previous studies on the SCZ have described the positive health economic impact per patient treated (25); staff experiences, including the benefits of collocation despite a lack of administrative infrastructure to support inter-organisational working (26, 27), and service utilisation, describing an uptake in preventive health (smoking cessation and vaccinations) and how nearly a third of patients saw the family support officer (28). These findings are complemented by the work presented here, a qualitative exploration of parents' experiences of using the service. The data were analysed and presented through an *a priori* framework that allowed us to identify and describe the key elements that contribute to the overall acceptability (29), including their understanding of the SCZ, its accessibility, the degree of cultural alignment, and their perceptions of its effectiveness.

## 2 Methods

### 2.1 Study design

The study used focus groups conducted with families that had used the SCZ. The data were analysed using a directed content approach to populate Sekhon's theoretical framework of acceptability (TFA) (29, 30). Ethical approval was granted by the London–Harrow Research Ethics Committee (25/PR/0168).

### 2.2 Setting/recruitment

The SCZ is based in the Sparkbrook & Balsall Heath East ward in Birmingham, UK. It is the second most populous ward in the city, with a superdiverse, young population that has the second highest level of deprivation in the city, with high unemployment rates and high levels of universal needs around housing, food, and clothing (31). It is disproportionately affected by childhood obesity and child criminal and sexual exploitation and has one of the highest levels of infant mortality in England (31).

Our recent study used data routinely collected via a dashboard sitting on the Egton Medical Information Systems (EMIS) system to explore patient activity and service uptake of the SCZ from February 2022 to October 2024 (28). From this, it was determined that there were approximately 14,000 CYP (i.e., <16 years old) registered to Sparkbrook PCN's eight practices. In summary, some 46% of those attending were female; 14% were aged <12 months, 34% were aged 12–59 months, and 59% were aged >60 months. There were limited data on ethnicity, but the largest proportion of patients were Pakistani (57%). The Index of Multiple Deprivation (IMD) scores of patients available for the total cohort were overwhelmingly in the most deprived quintile (89%) (28). Patients (over the age of 14 years) and/or their parents or guardians who had used the SCZ were eligible for inclusion in the study. The SCZ liaison officer (CL) approached potential participants in person using convenience sampling, i.e., those attending the clinic during the recruitment period between June and December 2025 (32). All those interested were provided with a participant information sheet, allowed to ask questions about their participation, and then asked to provide informed consent before the focus group commenced.

### 2.3 Data collection

Focus groups were conducted in August 2025 and November 2025, led by the first author (IL), a white man with a university education, trained and experienced in qualitative research, with a focus on equitable health service delivery. He led the previous work evaluating the SCZ (25–28) but was unknown to participants prior to the study. A topic guide was used, informed by the existing literature and previous qualitative work conducted with SCZ staff (26, 27). The questions included are as follows: How participants became aware of the service?, What they understood of its structure and its overall accessibility?, and Which elements worked well or might be improved? A summary of the topic guide is available in Supplementary File 2. The focus groups were conducted in English at two community centres in the same locale as the SCZ clinic; an interpreter was present at each centre, translating from Somali (Focus group 1) and Urdu (Focus group 2). The groups were digitally audio-recorded and transcribed verbatim by an authorised transcription service, and the data were managed using NVivo.

### 2.4 Data analysis

**Figure 2:**
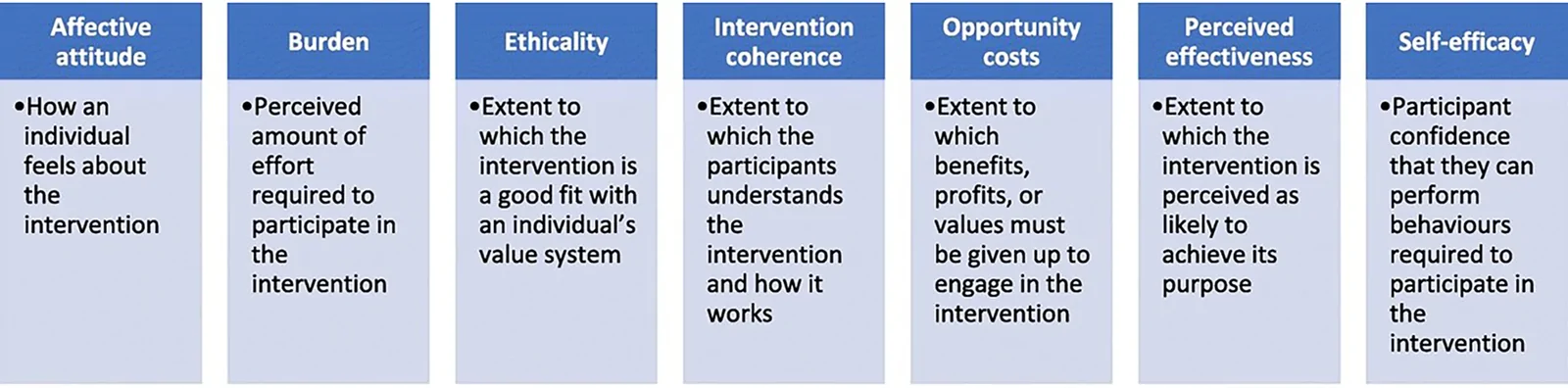
Summary of Sekhon’s theoretical framework of acceptability.

Analysis was informed by the TFA developed by Sekhon et al. to explore acceptability of healthcare interventions from the perspectives of providers and patients (29). It consists of seven domains: affective attitude, burden, ethicality, intervention coherence, opportunity costs, perceived effectiveness, and self-efficacy (see Figure 2). Together they describe whether those receiving (or delivering) an intervention consider it to be appropriate based on anticipated or experiential cognitive and emotional responses (29). Its structured approach allows for the isolation of the various components and attributes of acceptability and helps predict sustained engagement and effectiveness. It has been successfully used in a range of settings and circumstances, including the treatment of HIV (33), breast cancer (34), diabetes (35), and mental health (36).

The first and last authors (IL, CB) independently coded each transcript, fitting the data within each of the relevant domains of the TFA using a directed content analysis, following the unconstrained matrix approach suggested by Elo and Kyngäs to allow for the development and inclusion of emergent constructs or sub-constructs within the established domains of the framework (30, 37). The data were first allocated within the appropriate domain of the TFA; emerging themes or “constructs” within the domains were then iteratively refined and agreed upon by the two primary coders. Ultimately, the data were allocated within three of the TFA’s domains: *Intervention coherence*, *Burden*, and *Perceived effectiveness.* We retained the definition of a fourth TFA domain, “Ethicality”—summarily described by Sekhon as “the extent to which the intervention is a good fit with the individual’s value system”—but renamed it “Cultural sensitivity”, which we felt better reflected the content of the domain in this instance. The remaining domains were unused, as no relevant data emerged from the focus groups (29). The final allocation of data within each domain and construct was consensually agreed upon by all authors.

## 3 Results

### 3.1 Participant characteristics

The intention was to run up to four focus groups with parents (or caregivers) and children (>14), but only two were convened, consisting solely of parents. Recruitment proved challenging, and although there was no record of the number of patients/families approached, reasons for declining participation included a lack of ability to travel to the focus group venue and caring and other familial responsibilities (the challenges of recruiting underserved groups for research studies are further explored in the Strengths and Limitations section). All those who agreed to participate in the focus groups did so, apart from one parent who did not provide a reason for their non-attendance. The first focus group, consisting of six parents (plus one interpreter), lasted 47 min; the second focus group, consisting of two parents (plus an interpreter), lasted 24 min. Because of the small number of participants and their potential identifiability, we have minimised the description of each to gender and family characteristics (i.e., number of children), as further detail would risk compromising their confidentiality, as per the ethical approvals. There were five mothers and three fathers, with a maximum of five children and a minimum of two. One participant did not disclose the total number of children (see Table 1 for summary).

**Table 1:** Participants’ characteristics.

Participant ID	Relationship to patient	Number of children
P01	Father	Five
P02	Mother	Three
P03	Mother	Two
P04	Mother	Four
P05	Father	Two
P06	Father	Undisclosed
P07	Mother	Five
P08	Mother	Two

### 3.2 Qualitative findings

Below, we present the qualitative findings within four domains as defined within the TFA and exemplar quotes describing the emergent constructs within each (29). The domains, their definitions, and the constructs that emerged within each are summarised in Table 2. These are explored in the following, where the quotes are identified by their participant ID code and relationship to the patient.

**Table 2:** Summary of findings.

Domain	Definition	Emerging construct	Participants’ responses
1. Intervention coherence	How do participants feel about the intervention, and how well are its constructs understood?	1.1 Awareness	Participants learned about the SCZ from a range of sources, including promotional leaflets, GPs, and other parents
		1.2 Understanding of structure	Although participants knew the service was multidisciplinary, they reported uncertainty about the precise specialty and role of those they were seeing Some participants reported uncertainty amongst their GPs about who the SCZ was for
2. Burden	Perceived amount of effort required to use the intervention	2.1 Access	Having to seek a referral from their (busy) GP caused some delay in access, but once referred, waits for an SCZ appointment were relatively short The use of community locations supported ease of access
3. Cultural sensitivity	How well intervention align with individual beliefs and values?	3.1 Trusting relationships with providers	Participants felt able to describe not only their child’s medical symptoms but also their broader circumstances
		3.2 Language	Limitations in the availability of interpreters or bilingual staff led to linguistic barriers to understanding
4. Perceived effectiveness	How well the intervention can meet user needs and preferences?	4.1 Extended consultation time	Participants felt they had time to describe the full range of clinical and social needs
		4.2 Address complexity	Participants felt that the SCZ was capable of addressing the needs of neurodiverse or multimorbid patients
		4.3 Capable of advocacy	Participants appreciated how SCZ staff were able to advocate for them with local council authorities and schools

#### 3.2.1 Intervention coherence

Intervention coherence describes how far an intervention and its constituent elements are understood by those using it (29). Participants described this within three constructs: how they became aware of the SCZ, their understanding of its structure in relation to its multi-disciplinarity, and the processes involved in follow-up.

##### 3.2.1.1 Awareness

The broad understanding of what the SCZ consisted of was derived from a variety of sources, including their GP, leaflets at a local “play and stay” group, word of mouth from other parents, local events at a range of faith centres (including both churches and mosques), and the SCZ’s outreach nurse who worked with local schools. A full explanation of what the SCZ delivered was provided when they attended (“…when we were at Children Zone, the entire process was explained to us…” *P06, Father*). This range of methods appeared to be broadly effective in raising awareness amongst target populations, although, as one father described, the same level of awareness was not shared by all GPs, some of whom failed to understand what the SCZ could provide:

“ …I feel like my GP sees them as a downgraded version of getting help or something…, I told the issues that I feel like he’s got autism, he’s got this—but they refused for me to see the Children Zone, and they said, ‘You need to see the doctor for this.’” *P06, Father*

##### 3.2.1.2 Understanding of structure

Parents recognised the value of the clinical expertise they received [“the (Pediatric consultant) we saw at the Children Zone was extremely knowledgeable” *P06, Father*] and described their appreciation of the practical advice and supporting information they received from the family support officer:

“As soon as [family support officer]…straight away she asked me everything, and then she was, ‘Okay [P03], I’m going to send you this, this, this, this, this…’” *P03, Mother*

The core service offer of the SCZ was provided by a range of clinical staff, including practice nurses, GPs, and consultant paediatricians, alongside family support officers from GreenSquareAccord. Despite participants understanding and appreciating the multidisciplinary nature of the SCZ and multiple consultations during a single visit, it left some unsure of who exactly they had seen and which organisation they were from:

“Yeah. He’d walked us into the other room, and I think she did introduce who she was. But with him I just kept wondering afterwards was he a doctor, or was he something else?” *P02, Mother*

##### 3.2.1.3 Follow-up by SCZ

A number of participants described their lack of understanding of follow-up procedures (if any) following their attendance (“…I’ve had no contact with them after that…” *P02*). For some who were managing at least one other child in addition to the patient, the follow-up pathways were unclear, and they were unsure as to whether there would be a follow-up appointment. As a result, some we spoke to would have preferred greater clarity as to the next steps:

“I felt like…especially when we’re struggling with kids—that we can’t understand…it would have been nice to say, ‘Look, six weeks’ time, or even in a four to five months’ time we’ll give you a follow-up appointment, just to see how you’re getting on and what you need to do, and what you’ve tried and what you haven’t tried.’…” *P03, Mother*

#### 3.2.2 Burden

Burden describes the perceived amount of effort required to use the SCZ (29), and this was described by participants in terms of the efforts required to access the SCZ.

##### 3.2.2.1 Access

All those wishing to access the SCZ had to be referred by their GP. For those who learned of the SCZ during that consultation, this was a straightforward process (“…my GP referred me because of the school was causing me distress about my daughter’s attendance…” *P03, Mother)*. Still, for those who heard about the SCZ elsewhere, they found the extra step of having to secure a referral from their GP frustrating, preferring to arrange a consultation directly with the SCZ:

“It was really difficult to get an appointment…they said ‘phone reception and ask for ‘the Children’s Zone’, but they said, ‘No, you have to see the GP first, and then they will…[refer you]’…” *P02, Mother*

Once referred, however, parents described what they felt was a relatively short wait of 1 to 2 weeks for their appointment at the SCZ [“Yeah, so (once) I was referred to them. Got my appointment really quick, it wasn’t a long wait” *P03, Mother*]. They were also satisfied that the location of the clinics was readily accessible on foot, by car, or public transport (“…if you have car it’s easier, but if the worse the bus, or you can walk.” *P07, Mother*).

#### 3.2.3 Cultural sensitivity

The domain of cultural sensitivity describes how well the intervention aligns with the beliefs and values of the individuals using it (29). Participants described this in terms of the understanding relationships they developed with SCZ providers and the challenges of talking in their preferred language.

##### 3.2.3.1 Trusting relationships

Parents repeatedly described feeling understood by SCZ staff and that the connection they made with them felt personal and genuine. As this exchange within the first focus group demonstrates:

*P03 (Mother):* I think it's more that they build a rapport.

*P02 (Mother):* They don't see you like a number—it's really personalised.

*P01 (Father):* The thing is they don't just do the job—you know what I mean? They listen you, they care about you—you know? They give you the time—treat you like you're a person they try to understand your problem…

This perception was supported by the willingness of SCZ staff to not only address the issues facing their child but also to hear and understand the broader challenges they faced as a parent:

“Sometimes, I was so overwhelmed so just talking to her, she was not in rush at all. She used to listen me, sometime half an hour, 45 min—she was very patient, so helpful, you know what I mean?” *P01, Father*

##### 3.2.3.2 Language

The diversity of local populations and the number of people for whom English was not spoken as a first or preferred language led to SCZ to utilise an interpreter as part of its service (both in person and via a remote service available on some SCZ staff members’ mobile phones). However, constraints of capacity meant they were not available for every consultation, nor were they capable of speaking every language and dialect required (31). One participant explained (through her interpreter) that she would try to take a friend or relative with her to consultations; otherwise, her understanding was limited:

“So [*P08’s]* daughter has actually got Down’s Syndrome, so her doctor referred her to Sparkbrook…and [*P08*] is just saying that she doesn’t really get to understand a lot, because she doesn’t understand English much… if someone is able to go with her to translate it there then she will understand better, but otherwise if she has to go alone then it’s just whatever she can make of it.” *P08, Mother via her interpreter*

#### 3.2.4 Perceived effectiveness

The domain of perceived effectiveness refers to how well the intervention can meet user needs and preferences (29). Specific to the SCZ, three key elements emerged: the extended consultation time, the SCZ's ability to address complex needs, and its capacity to advocate for both patients and their families in a range of settings.

##### 3.2.4.1 Extended consultation time

Participants repeatedly described how the extended consultation time offered by the SCZ allowed time to talk through the key issues relating to the child but also the broader challenges they faced as a family, in contrast to the shorter “single problem” consultation at their GP surgery:

“…I’m happy because the nurse…I take long time, I ask a lot of things—but our GP? When you have appointment, we must only *one* [symptom] talking about…I feel it’s good to get more help, more information.” *P07, Mother*

Patients felt able to discuss a variety of clinical and social challenges and appreciated how the provider was able to assimilate this broad range of information and produce a systematic plan in response:

“…you’re just spilling your guts out, they allowed you to do it all, and then…they picked on certain topics. So, it was like, ‘Okay, say everything that you need to say, and let’s take it step by step.’ And then it just went into bullet points, ‘Okay, you mentioned this, how should we cover that? What do you expect and what support you need?” ”*P03, Mother*

##### 3.2.4.2 Ability to address complexity

Parents described how staff at the SCZ were able to address complex conditions and contexts. For example, a parent with an autistic child described how the SCZ provided signposting to further information and practical strategies appropriate for an autistic child, an element missing from the watchful waiting adopted by their usual GP:

“…when I met the Children Zone they were…it’s like so much information, and they were giving you in regarding to food habits, toiletry, even brushing their teeth—because autistic kids completely different understanding—linking you to websites and stuff, online courses, things like that. So, there’s a lot to it compared to when you go to the doctor and say to you…‘Oh yeah, you’ve just got to monitor your child.’” *P03, Mother*

In another example, a father described through their interpreter how they had toured the NHS, unable to find the right help for a disabled daughter who was suffering with depression until they visited the SCZ, who were able to provide both the medical and social support they needed:

“Because he met a lot of professionals, and they didn’t help, the help that he needs to have about his daughter, because she is disabled, and they have low income, and also the daughter she has depression… But when he met [family support officer] she asked first thing, ‘Please don’t leave me in the middle of the situation, so help me.’ So she helped a lot, and also about the benefits, about the…that she needs physiotherapy…” *P05, Father* (via interpreter)

##### 3.2.4.3 Capable of advocacy

The ability of the family support officer to complement the clinical expertise of the SCZ was appreciated, particularly in providing the support they needed as parents to navigate what can be a complex health and welfare system. For example, one participant noted how the family support officer had successfully advocated on their behalf with the local authority, comparing them favourably to other advisory organisations:

“…she knows I was on a council [home] waiting list, and she’s having a meeting with them, even though she signed me up [for a home] she’s, ‘I’m going to your housing officer…just to have a look that they’ve covered every base in your application form.’—and that’s something nobody ever does. Even if you go to Citizens Advice you won’t get that…, she’s actually gone out of her way and said, ‘I’ll cover all these for you, you just concentrate on your kids…’” *P03, Mother*

On another occasion, the family support officer advocated for a family whose child was struggling at school, helping school staff better understand the social circumstances of the child:

“…she’s so good…she helped me with this while the school not understanding when I have explaining them that he’s going through that issue. But through Children Zone she went, she explained them, then she explained me, ‘That’s what I’m doing, don’t worry.’” *P01, Father*

## 4 Discussion

### 4.1 Summary of findings

The patient and their family’s voice is central to developing NMDTS that are appropriate and effective, and this work has used the TFA to provide a structured understanding of the acceptability of a pilot NMDT in Birmingham, UK. In summary, by domain, we found that in considering *Intervention coherence*, individuals became aware of the SCZ through a range of sources, understanding that it was multidisciplinary even if sometimes unsure of precisely the identity or the organisations of those they had spoken with; In *Burden*, parents described the benefits of local, collocated services and relatively short waits for an appointment once referred; in considering *Cultural sensitivity*, parents described how they felt understood by the staff in the SCZ; and finally, in *Perceived effectiveness*, they described the benefits of the extended consultation time, the SCZ's ability to deal with complex patients and social contexts, and the benefit of the advocacy it provided.

### 4.2 Specific findings

#### 4.2.1 Awareness

The SCZ used several strategies to raise awareness amongst patients and their families at community locations and events, including faith organisations and local schools, a strategy that has previously proved successful in encouraging engagement in underserved communities (38). The multidisciplinarity of the service was understood and welcomed by parents, although there appeared to be some confusion over the specialty of those they had seen. This consequence of MDT care has been recognised previously, where there is evidence it can reduce continuity of care, fragment relational continuity (39), and lead to a lack of understanding of the role of each of the (health)care providers they saw, or the identity of their main doctor (40). It is important that in future iterations of NMDTs for CYP, families remain aware of the structure and the identity of who they are consulting, given evidence that patients' service awareness can influence engagement and outcomes (39). The complexity of multi-agency MDTs can also cause uncertainty in the surrounding healthcare system, with one parent describing how their GP failed to understand the SCZ offer. If the NHS's move to NMDTs is to be successful, there needs to be engagement with, and support garnered from, the surrounding system (41).

#### 4.2.2 Burden

There are several recognised barriers to accessing primary care in underserved populations, including financial and temporal constraints, poor housing, domestic violence, or food poverty (42). Amongst these is the limited ability of families to travel independently (43); however, the SCZ's collocated, integrated service and community setting facilitated access for parents, echoing previous examples where collocation in local communities improved access and reduced no-shows (44, 45). Similar NMDT offers for CYP in Australia (46) and elsewhere in the United Kingdom described the benefits to attendance and care navigation of localised, integrated care (13).

Parents felt that the wait of a week or two for an appointment was acceptable, although there were concerns over the effort and delay of first seeking a referral from their GP, particularly in light of lengthening wait times for GP consultations (47). Such waits are the leading reason for people failing to contact their GP in the United Kingdom (48); in this context, there may be a role for online booking and triage tools to provide more direct access to future NMDTs (49, 50), with the proviso that digital connectivity and literacy are appropriately supported (51).

#### 4.2.3 Cultural sensitivity

Parents described developing trusting, personalised relationships with their care provider. In the United Kingdom, the Royal College of General Practice has described the value of this relationship-based care, establishing rapport and empathy with patients (52), and there is growing use of similar non-transactional approaches that prioritise trust and ongoing care in the United States (53). In the SCZ, this was supported by a consultation style that followed many of the principles of the “inner consultation model,” i.e., making a connection, summarising key points, and ensuring parents were aware of next steps (54). The parent–provider relationship was also supported by other recognised strategies, including a caring communication style and exhibiting a genuine interest in the patient (55). Building trust is needed to support engagement in underserved populations, where it is hindered by poor experiences with mainstream healthcare (43).

Despite the ability of SCZ staff to foster relationships with families from a range of backgrounds, familiar issues around interpretation and understanding were described (56). It is known that language barriers can compromise quality of care, patient safety, and health outcomes (57). Although the SCZ provided interpreters and employed multilingual members of staff, the local population speaks some 70 languages, presenting logistical issues witnessed elsewhere in UK primary care (58).

#### 4.2.4 Perceived effectiveness

Previous work has reported how additional time is required in complex consultations with underserved populations (59) and parents described how the extended consultation time in the SCZ allowed for discussion of their family’s broader and often complex medical and social needs. There is already evidence that longer consultations are associated with better health outcomes (60). The ability of parents in the SCZ to discuss an unlimited number of issues led to a more holistic understanding of the patient and their context, as well as the ability to address their broader social needs. This is in contrast to existing recommendations in the United Kingdom for one problem per appointment, despite evidence that shorter contact times lead to more frequent visits (61).

Many healthcare systems have GPs playing a primary role in identifying autism in children and subsequently managing their care (62). Despite this, GP training and experience in autism varies (63), with many employing the gatekeeping principles of “wait and see” (62). This chimes with the experience of parents of neurodiverse children before attending the SCZ and previous reports that the needs of neurodiverse children are dismissed, minimised, or otherwise unacknowledged by frontline healthcare professionals (64).

Parents were grateful for the advocacy provided by the family support officer amidst a complex UK welfare system that sees some £23 billion of benefits remaining unclaimed (65). This fits with evidence of the importance of care navigation in these settings and (19) and of advocacy in health and social care in the United Kingdom, which has now been formally recognised by the National Institute for Clinical Excellence guidance (66). This includes the ability of advocacy to support patients' voices and mobilise resources, including challenging decisions being made by local authorities (66). However, more work is needed to understand how advocates can most effectively be included within primary care teams (67).

### 4.3 Strengths and limitations

The work highlighted key advantages and potential challenges of delivering NMDTs to children, particularly those from underserved populations. Our work also demonstrated that underserved CYP and their families remain some of the hardest to recruit into research, echoing previous challenges seen in multiple High Income Countries (HICs), including the United States (68), Canada (69), Australia (70), and the United Kingdom (71). The broad barriers to research participation, such as awareness, understanding, and opportunity, are exacerbated for underserved CYP and their families (72), alongside competing responsibilities of work and caring for other family members that were cited in this study (73). Knowing this, we used recommended strategies, including employing a recruiter who had developed relationships with local communities (through their work for the SCZ), using local community venues, offering financial incentives in the form of a gift voucher (74), and involving our Patient and Public Involvement (PPI) panel to word the Patient Information Sheet. Although the lower-than-expected number of participants limits broader generalisability, many of the experiences they described were shared. The quality of the data was supported by regularly sharing and discussing the outputs of the analysis across the team (75); in addition, we have provided a detailed account of the research setting and the users of the SCZ and kept an audit trail of data collection and storage (76). Taken together, this means that, although limited, the data remain capable of offering valuable insight to inform similar service offers for CYP in equally diverse populations.

### 4.4 Conclusions

NMDTs are being rapidly introduced into health and social care in the United Kingdom despite a lack of corresponding evidence. This work offers valuable insight into parent perspectives of an NMDT for CYP in a diverse and economically disadvantaged ward in Birmingham. Parents appreciated the collocated and community-sited premises, the provision of relationship-based care, advocacy, and support for the clinical needs of their child and broader social circumstances. However, more work is needed with NMDTs of different designs and patient populations to understand the impact of different configurations. Meanwhile, our findings suggest that future iterations of similar services might consider more direct pathways involving online systems, ensure that the surrounding health system is aware of the service, and ensure that families understand the membership and roles of their care team and extended consultation times.

## Data Availability

The raw data supporting the conclusions of this article will be made available by the authors, upon reasonable request.
