# Supplementary material for: Experiences of families using an early example of a neighbourhood multidisciplinary care team for children and young people in Birmingham, UK: a qualitative exploration of acceptability
Source: Front Pediatr. 2026 Aug 17;14:1896606. doi: 10.3389/fped.2026.1896606 (PMC13527020; doi:10.3389/fped.2026.1896606)
Supplement: Supplementary file 1 [file Supplementaryfile1.docx]

**Supplementary File 1: Key components of the SCZ service**

**Table 1:** Summary of the key components of the SCZ care offer [1].

**Preventative health care**

- Smoking cessation advice (if there is a smoker in the household)
- Immunisation advice
- BMI calculated for CYP >2 years (and onward referral to a healthy eating/exercise programme when available/funded)
- An oral health check and a toothbrush pack (children who were amber/red on the screening check had support accessing a dentist if the child was not registered with one)
- Healthy Start vitamins for CYP <4 years and mothers with an infant
- Directed via SMS post-clinic to the NHS Healthier Together website (<https://www.what0-18.nhs.uk/>) to aid self-management of common conditions

**Clinical appointments**

- Clinic appointments are 25 minutes in length. GPs and paediatricians see all presentations except acute injuries (which are better managed in minor injury units or EDs)
- After taking time to discuss care plans with CYP and their families, clinicians were encouraged to provide written summaries of their care plans which were sent via SMS text for all CYP attending with asthma, eczema or constipation (following guidance from the UK’s National Institute for Health and Care Excellence NICE)
- Any child or family with social support needs are referred directly to the team’s family support worker
- Electronic consultations offering remote clinical advice to GPs working for the partner PCN

**Early help**

- Usually referred directly after the clinical appointment but can be referred directly to Early Help by GP, school
- One-hour slots
- Early intervention to prevent social issues for families escalating
- Early Help workers navigate issues for families with schools, health, the voluntary sector, sexual health services and many others to enable families to thrive
- Early Help provides a broad range of services for CYP and their families including counselling

1.	Bird, C., et al., *Early activity and impact of a neighbourhood multidisciplinary team that integrates health and social support for underserved children and young people in Birmingham, UK: an observational study.* BMJ Paediatrics Open, 2025. **9**(1): p. e003935.
