# Supplementary material for: Experiences of families using an early example of a neighbourhood multidisciplinary care team for children and young people in Birmingham, UK: a qualitative exploration of acceptability
Source: Front Pediatr. 2026 Aug 17;14:1896606. doi: 10.3389/fped.2026.1896606 (PMC13527020; doi:10.3389/fped.2026.1896606)
Supplement: Supplementary file 2 [file Supplementaryfile2.docx]

**Supplementary File 2 Summary interview topic guide**

*Introductory question*

- Can you all please tell me a bit about yourselves (for example, how long have you lived in your neighbourhood? How many children do you have?)

*Thinking about your awareness of the service…*

- How did you hear about the service?
- What did you understand about what it offered?
- Was the reason for your referral explained?

*Thinking about your consultation…*

- How easy was it to get to the premises and find the service?
- Who did you see?
- Were you signposted to other care providers within the building?

*Thinking of next steps…*

- Following your appointment, did you attend or were you directed to any other services

*Thinking of your overall experience…*

- How did you find the service provided by SCZ?
- What aspect went well or might be changed to make it easier using the service?
